# Multiple Myeloma with Intracytoplasmic Azurophilic Granules

**Published:** 2019-04-01

**Authors:** Alireza Sadeghi, Pardis Nematollahi, Azadeh Moghaddas, Ali Darakhshandeh

**Affiliations:** 1 Department of Medical Oncology and Hematology, Isfahan University of Medical Sciences, Isfahan, Iran; 2 Cancer Prevention Research Center, Isfahan University of Medical Sciences, Isfahan, Iran; 3 Department of Clinical Pharmacy, Isfahan University of Medical Sciences, Isfahan, Iran

**Keywords:** Multiple myeloma, Azurophilic granules, Plasma cell

## Abstract

Bone marrow examination plays an important role in the diagnosis of multiple myeloma. In some cases with multiple myeloma, marrow plasma cells with cytoplasmic inclusions are seen. In this study, a 46-year- old man was evaluated for multiple myeloma. In bone marrow aspiration, large intracytoplasmic azurophilic granules, resembling intracellular microorganisms were seen. IHC study demonstrated that these cells are CD138 positive. This is a rare histologic finding that usually results from the deposition of excess immunoglobulin.

## Introduction

 Multiple myeloma is a plasma cell neoplasia characterized by bone marrow infiltration with monoclonal plasma cells^1^. It is the second most common hematologic malignancy and accounts for 1.8% of all cancers and 17% of hematologic malignancy^2^. Clonal bone marrow plasma cell more than 10% or biopsy-proven soft tissue or bony plasmacytoma plus at least one organ-related damage are required for the diagnosis of multiple myeloma.

## Case presentation

 Here,* we* report a case of a 46-year-old man evaluated because of progressive bone pain and night sweats from 2 months ago. The past history and family history were not remarkable, except the history of appendectomy in childhood. Laboratory studies showed mild normochromic normocytic anemia (Hb: 10.1 g/dL), hypercalcemia (Ca: 13.1 mg/dL), and renal impairment (seum ceraetinin 1.8 g/dL). Serum protein electrophoresis and immunofixation showed a 7.6 g/dL IgG kappa monoclonal paraprotein. Wright-Giemsa–staining of bone marrow aspiration and biopsy showed hypercellular marrow with abnormal plasma cells that involved 50% of bone marrow.

Some of these plasma cells had intracytoplasmic large azurophilic granules that resemble intracellular microorganisms (Figures 1a and 1b).

**Figure 1a:**
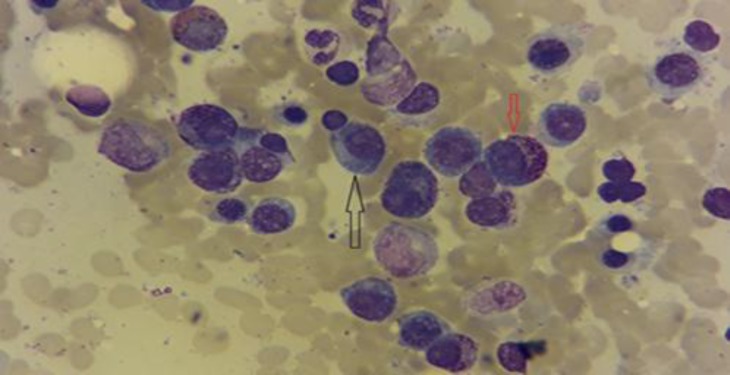
Plasma cells with eccentric nuclei and basophilic cytoplasm (black arrow)

**Figure 1b:**
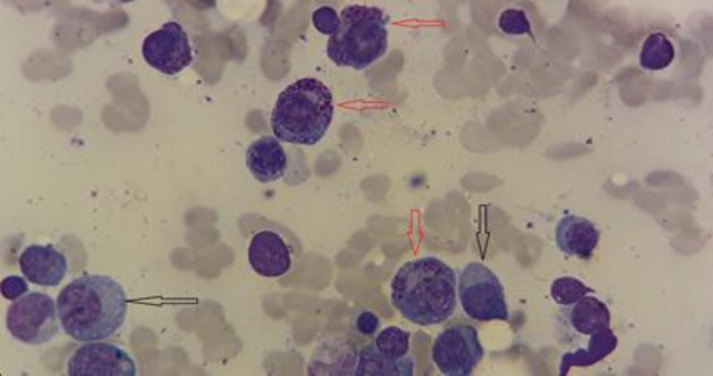
Abnormal plasma cells with numerous azurophilic granules (red arrow) (1000#)

Fixed paraffin-embedded bone marrow stained with CD 138 antibody using peroxidase-conjugated chromogen showed membrane staining with myeloma cells (Figures 2a and 2b).

The diagnosis of multiple myeloma was confirmed by the laboratory and pathologic findings, and then the patient was treated with a combination of bortezomib, cyclophosphamide and dexamethasone regimen. After four cycles of chemotherapy and achieving a very good partial response, the patient underwent autologous bone marrow transplantation. 

**Figure 2a:**
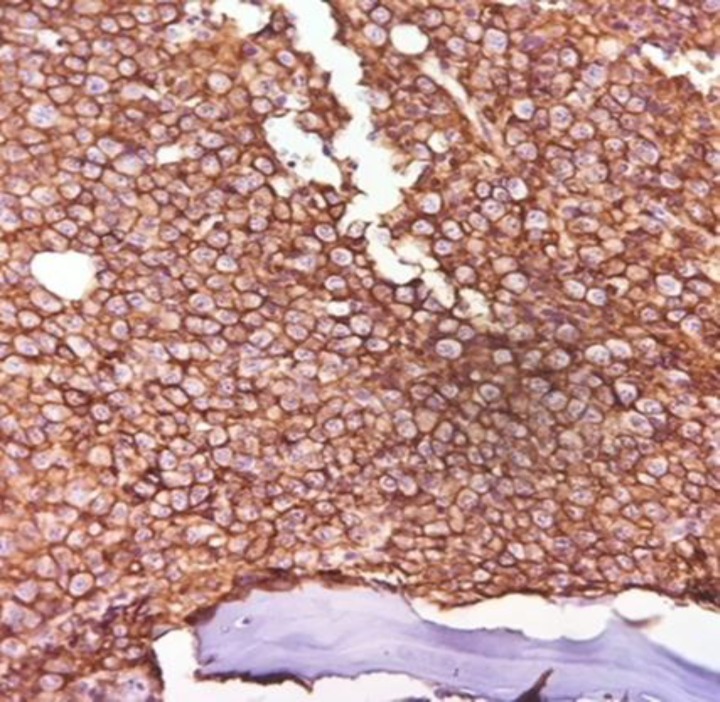
IHC staining for CD 138

**Figure 2b:**
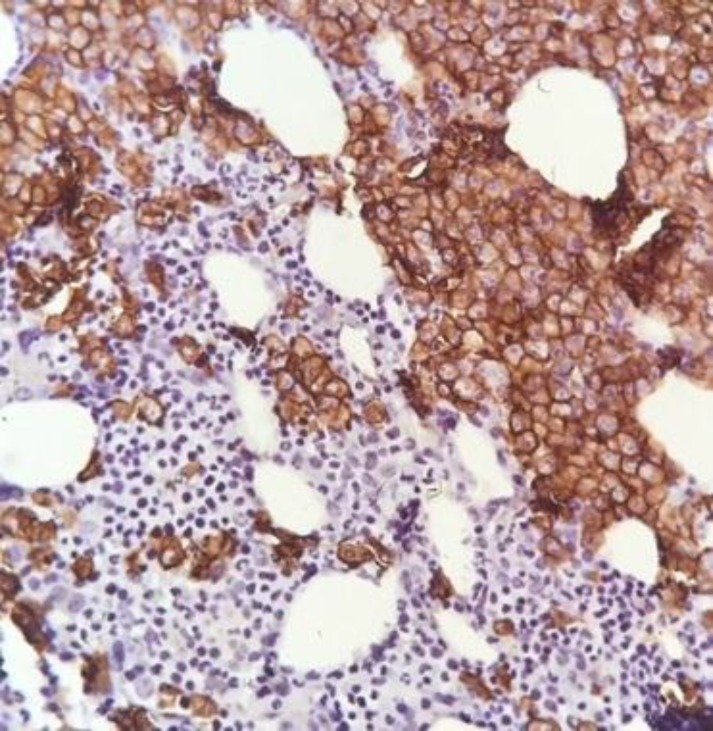
large sheets of myeloma cells (400#)

## Discussion

 Bone marrow study had a critical role in the diagnosis of multiple myeloma. Abnormal plasma cells are usually larger than reactive plasma cells which have *eccentric nuclei* and a perinuclear clear area^3^. In some patients, plasma cells may contain intracytoplasmic crystallized immunoglobulin, resulting in unusual findings such as Mott cells, Russell body, crystalline rode gaucher like cells and rarely azurophilic granules.

Intracytoplasmic azurophilic granules are a very rare histologic finding that usually result from deposition of excess immunoglobulin. Morphologically, these granules can mimic lysosomal storage disease or microorganism infection such as Leishmania^4^. In this situation, immunohistochemical studies have a critical role in excluding the differential diagnosis. 

Suzuki et al. reported that in patients with granule containing plasma cells, CD56 and CD49e expression are higher than other patients, but these granules did not affect survival^5^.
